# A simple and reliable method reducing sulfate to sulfide for multiple sulfur isotope analysis

**DOI:** 10.1002/rcm.8048

**Published:** 2018-01-30

**Authors:** Lei Geng, Joel Savarino, Clara A. Savarino, Nicolas Caillon, Pierre Cartigny, Shohei Hattori, Sakiko Ishino, Naohiro Yoshida

**Affiliations:** ^1^ Univ. Grenoble Alpes, CNRS, IRD Institut des Géosciences de l'Environnement IGE 38000 Grenoble France; ^2^ School of Chemistry Cardiff University Cardiff CF10 3AT UK; ^3^ Laboratoire de Géochimie des Isotopes Stables Institut de Physique du Globe de Paris, Sorbonne Paris Cité, Univ. Paris Diderot, UMR 7154 CNRS 75005 Paris France; ^4^ School of Materials and Chemical Technology Tokyo Institute of Technology 226‐8502 Yokohama Japan; ^5^ Earth‐Life Science Institute Tokyo Institute of Technology 152‐8551 Tokyo Japan

## Abstract

**Rationale:**

Precise analysis of four sulfur isotopes of sulfate in geological and environmental samples provides the means to extract unique information in wide geological contexts. Reduction of sulfate to sulfide is the first step to access such information. The conventional reduction method suffers from a cumbersome distillation system, long reaction time and large volume of the reducing solution. We present a new and simple method enabling the process of multiple samples at one time with a much reduced volume of reducing solution.

**Methods:**

One mL of reducing solution made of HI and NaH_2_PO_2_ was added to a septum glass tube with dry sulfate. The tube was heated at 124°C and the produced H_2_S was purged with inert gas (He or N_2_) through gas‐washing tubes and then collected by NaOH solution. The collected H_2_S was converted into Ag_2_S by adding AgNO_3_ solution and the co‐precipitated Ag_2_O was removed by adding a few drops of concentrated HNO_3_.

**Results:**

Within 2–3 h, a 100% yield was observed for samples with 0.2–2.5 μmol Na_2_SO_4_. The reduction rate was much slower for BaSO_4_ and a complete reduction was not observed. International sulfur reference materials, NBS‐127, SO‐5 and SO‐6, were processed with this method, and the measured against accepted δ^34^S values yielded a linear regression line which had a slope of 0.99 ± 0.01 and a R ^2^ value of 0.998.

**Conclusions:**

The new methodology is easy to handle and allows us to process multiple samples at a time. It has also demonstrated good reproducibility in terms of H_2_S yield and for further isotope analysis. It is thus a good alternative to the conventional manual method, especially when processing samples with limited amount of sulfate available.

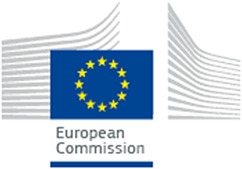

## 1 INTRODUCTION

Stable sulfur isotopes have been widely used to trace a range of biogeochemical processes.^1^ The discovery in 2000 of the mass‐independent isotopic fractionations of sulfur isotopes (S‐MIF) in sulfate and sulfide in Archean rocks^2^ showed the potential of the S‐MIF signals for tracking the oxygenation of the atmosphere 2.4 Gy ago,^3^ and the geochemical evolution of Mars.^4^ The S‐MIF signals in ice‐core sulfate have also been observed and demonstrated to be useful for tracking the sulfur cycle in today's stratosphere and they serve as a unique proxy of large volcanic eruptions that inject sulfur into the stratosphere and thus have global climate impacts.^5^, ^6^, ^7^, ^8^ Multiple sulfur isotope compositions can also help to constrain the oceanic sulfur cycle (e.g., 9, 10).

To access the S‐MIF signals, precise analysis of the four sulfur isotopes (^32^S, ^33^S, ^34^S and ^36^S) is necessary. The isotopic results are expressed as δ^3x^S = ^3x^R_sample_/^3x^R_CDT_ − 1, where x = 3, 4, and 6, and the δ values are calculated using the CDT standard. The S‐MIF values are then defined by:

$$\begin{array}{c} \Delta^{33}S=\delta^{33}S-\left[{\left(\delta^{34}S+1\right)}^{0.515}-1\right]\\ \Delta^{36}S=\delta^{33}S-\left[{\left(\delta^{34}S+1\right)}^{1.90}-1\right] \end{array}$$

The isotopic analysis is conventionally performed by reducing sulfate (SO_4_^2−^) to hydrogen sulfide (H_2_S), converting H_2_S into silver sulfide (Ag_2_S), and fluorinating Ag_2_S to sulfur hexafluoride (SF_6_) for isotopic composition analysis by isotope ratio mass spectrometry (IRMS).^2^, ^6^, ^11^, ^12^ The reduction from SO_4_^2−^ to H_2_S is mainly achieved by two different reducing agents: tin(II) (Sn^2+^) solutions and hydroiodic acid (HI)/hypophosphorous acid (H_3_PO_2_) mixtures.^13^, ^14^, ^15^ The Sn^2+^ solution is mainly applied to solid samples (e.g., minerals) with an optimum reaction temperature between 280 and 300°C, and the HI reducing solution can be applied to aqueous samples at 100–125°C.^14^ Currently, the most widely used reducing method in sulfur isotope geochemistry follows the reducing agent recipe (500 mL concentrated HI, 816 mL concentrated HCl, and 245 mL 50% H_3_PO_2_) of Thode et al.,^16^ and uses a distillation apparatus similar to that described in Forrest and Newman.^17^

In the reducing solution of Thode et al,^16^ high concentrations of HI seem to be the most important component of the reducing agent for complete sulfate reduction, and the presence of H_3_PO_2_ or NaH_2_PO_2_ increases the reduction speed by maintaining a high hydroiodic acid to iodine ratio which is one of the factors favoring the reduction.^14^, ^18^ HCl is only of secondary importance and its presence is suggested to increase the acidity and volume, and reduce the use of relatively expensive HI.^13^, ^19^ However, Gustafsson^20^ found the presence of water to be detrimental for the reduction because water tends to dilute and thus lower the concentration of HI, and at lower HI concentration, side products (*viz*, SO_2_ and elemental S) will be formed.^18^ In this regard, mixing 50% H_3_PO_2_ and concentrated HCl with the concentrated HI may have drawbacks for the reduction efficiency, because both H_3_PO_2_ (50%) and concentrated HCl (37%) contain more than 50% water by weight. To avoid additional water in the reducing solution, the H_3_PO_2_ can be replaced with dry NaH_2_PO_2_ salt, and HCl can be omitted. Gustafsson^20^ and Davis and Lindstrom^18^ have used a reducing solution containing only HI (57%) and NaH_2_PO_2_ salt, and found a good reduction yield. In particular, Davis and Lindstrom^18^ found that the optimum composition of the reducing solution for complete and fast sulfate reduction is 0.13 g NaH_2_PO_2_ in 1 mL HI (57%). In these studies, aqueous sulfate samples were processed and a cumbersome distillation apparatus was used.

In summary, it seems that the best composition of the reducing solution would be a mixture of 0.13 g NaH_2_PO_2_ in 1 mL HI (57%), and the amount of water in the reduction experiment should be limited. The latter requirement suggests dry sulfate samples are a better choice as the starting material. Typically, barium sulfate (BaSO_4_) is the preferred sulfate form for the four‐sulfur isotopes analysis because it is the natural form found in major geological samples or can be readily prepared from natural samples containing soluble sulfate (e.g., sea water) by precipitation with excess BaCl_2_ solution. BaSO_4_ has very low solubility (≈ 0.02 mg/L at 20°C) and this may inhibit the reaction efficiency and speed, especially when the volume of the reducing solution is small. Alternatively, soluble sulfate in natural samples can also be extracted and purified by other methods such as using an ion‐exchange resin^21^ and this can yield dry Na_2_SO_4_ by evaporating the eluent. We thus conducted tests with both BaSO_4_ and Na_2_SO_4_ to explore the reaction efficiency of the reduction process with respect to different sulfate forms. In this report, we present a series of experiments where we used a reducing solution comprising NaH_2_PO_2_ and HI (57% by weight) to process dry sulfate samples (both Na_2_SO_4_ and BaSO_4_). To avoid the cumbersome distillation apparatus, we tested a simple flow system with only sealed glass tubes connected by PEEK tubes and explored the possibility of processing multiple samples at one time. The reproducibility for H_2_S yield and for further sulfur isotope analysis is reported.

## 2 EXPERIMENTAL

### 2.1 Reagents

The new reducing solution was made of 100 mL concentrated hydriodic acid (HI, 57% by weight) and 13 g sodium hypophosphite (NaH_2_PO_2_). The reducing solution was prepared in a fume hood, where 100 mL HI and 13 g NaH_2_PO_2_ were added to a flask. The flask was placed on a hot plate magnetic stirrer and a magnetic stir bar was used to mix the liquid and the salt. Because HI is easily oxidized by O_2_, helium (He) or another inert gas stream (e.g., N_2_) was introduced by a PEEK tube into the flask to purge the mixture. While purging with He, the hot plate temperature was set at 130°C. The flask was heated at 130°C for at least 1 h to reduce any sulfur compounds into H_2_S (that was flushed away from the reagents) and to reduce traces of I_2_ (in the form of I_3_^−^) into I^−^ by NaH_2_PO_2_. The solution started with a deep color (I_3_^−^) and became colorless with time. After being heated for 1 h, the solution was allowed to cool down under the He stream and then stored in a sealed brown bottle. The reducing solution may become oxidized over time; this is indicated by a light yellow color, which may become deeper depending on the degree of oxidation.

Different from what can be found in the literature, in this study we used sodium hydroxide (NaOH, 0.1 M) as the trapping solution to collect the reduction product H_2_S. Conventional trapping solutions, cadmium (or zinc) acetate (Cd(CH_3_CO_2_)_2_, 0.1 M) and/or silver nitrate (AgNO_3_, 0.01 M) were also investigated, and the results were compared with that from the NaOH trapping solution. As detailed below, using NaOH as the trapping solution allows direct quantification of the sulfur concentration by UV absorption spectroscopy, which is faster and more reliable than gravimetric techniques.

### 2.2 Apparatus

**Figure 1:**
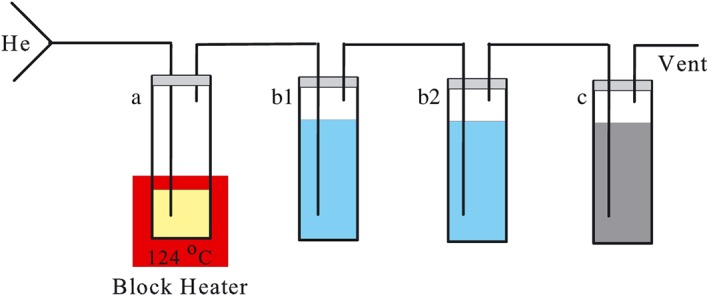
Sketch of the reduction train. a: block heater and the reduction tube; b1 & b2: gas washing tubes; c: H_2_S collection tube [Color figure can be viewed at http://wileyonlinelibrary.com]

The reduction train is sketched in Figure 1. The main parts of the apparatus are four 15‐mL glass tubes each with a nitrile/PTFE septum and a block heater. Reaction tube 'a', two gas washing tube 'b1' and 'b2' and the collection tube 'c' were connected with PEEK tubes (1/16" ID) directly through the septum. Alternatively, a drying cartridge filled with potassium perchlorate (KClO_4_) and a cryogenic trap (whose internal temperature can be controlled between −200°C and −80°C) can be placed between the trap 'b2' and the collection tube to test the possibility of using pure H_2_S as the working gas for isotope analysis. The drying cartridge and the cryogenic trap allow us to purify H_2_S without any loss. The dry sulfate sample (i.e. Na_2_SO_4_) and 1 mL reducing solution were introduced into glass tube 'a', which was placed on a block heater and purged with a He flow for 20 minutes before turning the heater to a temperature of 124°C. The purge before the heating stage is necessary to remove traces of I_2_, especially when the reducing solution has a light yellow appearance over time due to slight oxidation.

**Figure 2:**
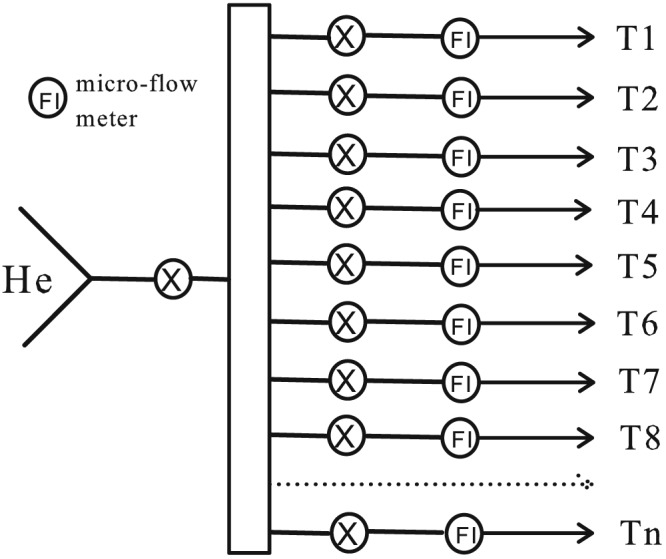
Sketch of the flow system containing multiple reaction trains. "T1...Tn" indicate the reduction trains assembled

The He gas was supplied from a tank. In practice, we used a home‐made flow distributor to distribute the He gas to eight reaction train flows, as shown in Figure 2. Each flow was then guided to an individual reduction train, and the flow rate (~2 mL/min) of each reduction train was controlled by a micro‐flow meter (ref: P‐446, IDEX Health & Science, Sainte‐Foy‐La‐Grande, France). In this way, multiple samples can be processed simultaneously.

### 2.3 Testing samples

We used the above‐mentioned reducing solution and apparatus to process dry sulfate samples in the forms of barium sulfate (BaSO_4_) and sodium sulfate (Na_2_SO_4_). The Na_2_SO_4_ samples were prepared from a 1 mM Na_2_SO_4_ solution (0.142 g Na_2_SO_4_ in 1 L Milli‐Q water; Millipore SAS, Molsheim, France), and then the desired volume of the Na_2_SO_4_ solution (e.g., 0.2 or 0.5 mL, equivalent to 0.2 or 0.5 μmol SO_4_^2−^) was added to a pre‐cleaned reaction tube. The reaction tube was allowed to completely dry in a 100°C oven, and the sample was then stored for later use.

In order to prepare the BaSO_4_ samples, the desired volume (e.g., 0.2 or 0.5 mL) of the 1 mM Na_2_SO_4_ solution was added to the reaction tube, and a drop of 1 M HCl solution was then added to remove any carbonate in the solution. After storage overnight, a drop of 0.1 M BaCl_2_ solution was added to the reaction tube to precipitate BaSO_4_. After the BaSO_4_ had precipitated, the samples were divided into two sets, which were then processed differently. One set of BaSO_4_ samples was dried completely in an oven at 100°C, so the dry samples contain BaSO_4_, BaCl_2_ and NaCl. We termed this set of samples BaSO_4_‐EB (BaSO_4_ with excess BaCl_2_). The other set of BaSO_4_ samples was centrifuged and the supernatant was removed. The remained solids were rinsed with Milli‐Q water and then separated from the rinsing water by centrifuging. This step was repeated three times before the sample was placed in the oven to dry. This set of samples was termed P‐BaSO_4_ (pure BaSO_4_).

In addition, international reference materials (in the form of BaSO_4_), IAEA‐SO‐5 (δ^34^S = (0.5 ± 0.2) ‰), IAEA‐SO‐6 (δ^34^S = (−34.1 ± 0.2) ‰) and NBS‐127 (δ^34^S = (20.3 ± 0.4) ‰) were prepared by weighing ~ 0.5 mg of the BaSO_4_ standards into reaction tubes. After reduction, these samples were further converted into SF_6_ for isotope analysis. We note that the reference materials were not weighed precisely because of the capability of our balance (0.1 mg precision). However, the purpose of processing these samples is to test potential sulfur isotope fractionation during the reduction, rather than to assess the reduction yield (which can be assessed from the samples made from drying Na_2_SO_4_ solution with accurate measurement of sulfur content, or precipitating BaSO_4_ from the same Na_2_SO_4_ solution).

### 2.4 Quantification

The yield of the reduction from sulfate (SO_4_^2−^) to sulfide (S^2−^) can be directly assessed by determining the quantity of H_2_S collected in the NaOH trapping solution. Hydrogen sulfide (H_2_S) solution is known to absorb UV light with a peak absorbance at 230 nm.^22^, ^23^ Guenther et al^22^ have shown that in alkaline solutions with pH >8, H_2_S is present nearly 100% in the form of the bisulfide ion (HS^−^), and they found that at pH ~8, UV determination of HS^−^ yields are accurate because precise estimates of total sulfide concentration in the solution can be achieved. Thus, with NaOH as the trapping solution, the yield of the reduction can be directly assessed by measuring HS^−^ in the solution with optical methods. In comparison, the conventional trapping solution (cadmium acetate or silver nitrate) collects H_2_S as a precipitate, which makes it difficult to directly quantify the reduction yield.

In this study, we used a UV spectrophotometer (model 6850; Jenway, Stone, UK*)* to determine the concentration of H_2_S in the NaOH trapping solution. The calibration standards were made by mixing sodium sulfide nonahydrate (Na_2_Sˑ9H_2_O, >99.99% purity; Sigma‐Aldrich, St Louis, MO, USA) with 0.1 M NaOH solution. A few crystals of Na_2_Sˑ9H_2_O were quickly rinsed on Kimwipes® disposable wipers to remove surface oxidation products, dried and weighed directly. A stock solution of 0.01 M HS^−^ was made by mixing 0.0125 g of pre‐cleaned Na_2_Sˑ9H_2_O in 5 mL 0.1 M NaOH solution. A set of working standards, 0.0 μM, 20 μM, 50 μM and 100 μM, was then made by diluting 0, 0.02, 0.05 and 0.1 mL of the stock solution into the required volume of 0.1 M NaOH to obtain a 10‐mL standard solution. The stock solution should be stored in a sealed brown bottle and flushed with He before storage, since sulfide is easily oxidized by O_2_ once in contact with air. Even when the stock solution was flushed before storage, we noticed significant loss of sulfide after 2–3 days. Guenther et al^22^ made the stock solution in a glass aspirator bottle purged with N_2_, and stated that the solution should be stable for about 1–2 weeks. In practice, we prepared a fresh stock solution once every 2 days, and working standards every day.

### 2.5 Procedure

Prior to the reduction, all glassware, caps, septum and PEEK tubes were cleaned with Milli‐Q water. The PEEK tubes have to be flushed to ensure that there is no water left inside them; otherwise the water will block the flow of the carrier gas in the reduction line.

In a fume hood, 1 mL of reducing solution was added to a pre‐prepared reaction tube to a known amount of dry sulfate. In the reaction tube, the reducing solution was purged with He for 20 min at room temperature to remove any I_2_ and O_2_. The gas washing tubes ('b1' and 'b2' in Figure 1) and the collection tube ('c' in Figure 1) were prepared by adding 12 mL Milli‐Q water and 12 mL 0.1 M NaOH, respectively. After the reducing solution had been purged for 20 min, the reduction train was assembled (Figure 1) and the reaction tube was placed in the block heater and heated at 124°C. At lower temperatures the reduction speed will be slow, while if the temperature is too high, an excessive amount of phosphine (PH_3_) will be produced from the decomposition of NaH_2_PO_2_.^14^ For the alternative setup, the drying agent was in‐line with the cryogenic system, and the latter was set at −200°C to trap the reaction products. When the reaction was over, the temperature of the cryogenic trap was raised to −120°C when the produced H_2_S was released and trapped in the collection tube.

The collection tube was removed from the reduction train after the reaction was complete. The concentration of H_2_S in the trapping solution was first measured by UV spectrophotometry as described in section 2.4, in order to assess the yield. Then 1 mL of 0.01 M AgNO_3_ was added to the collection solution to precipitate Ag_2_S and Ag_2_O. After gentle shaking, a few drops of concentrated HNO_3_ (68%) were added to the suspension. Following thorough shaking, the Ag_2_O dissolved and only Ag_2_S remained in the solid phase. The tube was allowed to settle, and a plastic laboratory dropper was used to remove the supernatant. The solid was then rinsed three times with Milli‐Q water, transferred to an aluminum boat and dried prior to fluorination.

### 2.6 Isotope analysis

To explore potential sulfur isotope fractionation during the reduction, we processed the international sulfate reference materials IAEA‐SO‐5, IAEA‐SO‐6 and NBS‐127, following the procedure mentioned in section 2.5. The reference materials were weighed, and approximately 0.5 mg was added to the reaction tube. The reaction was stopped after ~5 h.

After being converted into Ag_2_S as described in section 2.5, the reference materials were transported to the Stable Isotope Geochemistry Laboratory at IPG‐Paris (Institut de Physique du Globe, Paris, France) for sulfur isotope analysis. At IPG, the samples were dried, transferred to an aluminum boat and then weighed. Due to the small quantity (~0.3 mg Ag_2_S or less) of the sample, we found it is very difficult to transfer the dry Ag_2_S from the collection tube to the Al boat. As an alternative, we transferred the solid together with a small amount of water from the tube to the Al boat, and then dried the samples. Under these circumstances we found that, after drying, the inside wall of the Al boat became light‐brown in color, and the mass of the dried Al boat plus the sample exceeded the sum of the sample and the Al boat, indicating the gain of extra mass during the drying process. This is probably due to the development of a thin layer of Al_2_O_3_ on the Al metal surface when Al contacts with water at the drying temperature (70°C). This is consistent with the observation that, after drying an Al boat with Milli‐Q water at 70°C, a brown layer was formed on the inner surface of the Al boat and the mass of the Al boat was increased. Nevertheless, the fluorination yields and the sulfur isotopic analysis results suggested this influences neither the fluorination procedure nor the isotopic composition.

The dried Ag_2_S samples were fluorinated in nickel bombs under approximately 37 kPa of fluorine gas (F_2_) at 250°C overnight. The evolved SF_6_ was purified cryogenically and then by gas chromatography. Because of the small amount of samples (<0.5 mg Ag_2_S), a microvolume cold finger of an isotope ratio mass spectrometer (MAT 253; Thermo Scientific, Bremen, Germany) working in dual‐inlet mode was used to concentrate the sample gas for isotope analysis.^24^ The analytical uncertainty (1σ) for the instrument was 0.25‰ for δ^34^S values, 0.010‰ for Δ^33^S and 0.062‰ for Δ^36^S obtained by replicate analysis (*N* = 4) of IAEA‐S‐1 over a period of 4 weeks (once a week) when the processed sulfate standards were also measured for sulfur isotopic composition.

## 3 RESULTS AND DISCUSSION

### 3.1 H_2_S collection agents

The reduction product, H_2_S, has to be collected and converted into Ag_2_S before fluorination for isotope analysis. As mentioned above, Cd(CH_3_CO_2_)_2_ 16, 17 and AgNO_3_ 13 have both been shown to be able to efficiently trap H_2_S by forming CdS and Ag_2_S precipitates, respectively. The CdS precipitate is further converted into Ag_2_S by adding AgNO_3_ solution.^16^, ^17^

The conventional reducing solution commonly contains phosphorous acid (H_3_PO_3_) or hypophosphorous acid (H_3_PO_2_),^13^ and phosphine (PH_3_) is produced when the reducing solution is heated.^18^ Once PH_3_ comes in contact with AgNO_3_, it reduces Ag^+^ to Ag^0^ and this leads to excess precipitate in addition to Ag_2_S.^17^ To prevent this, Thode et al^16^ and Forrest et al^17^ used Cd(CH_3_CO_2_)_2_ as the trapping solution. In particular, Forrest et al^17^ flushed the Cd(CH_3_CO_2_)_2_ solution with N_2_ for 15 min after the CdS precipitated and prior to adding AgNO_3_. This step was found to effectively remove PH_3_ and thus no excess precipitate formed. However, Arnold et al^13^ found that when using AgNO_3_ as the trapping solution, the excess Ag precipitate in the trap is not detrimental to the final isotope analysis of the sulfur content after fluorination. Because of this, AgNO_3_ appears to be the better reagent for the collection of H_2_S, given the environmentally toxic nature of Cd^2+^.

In this study, we first employed 0.01 M AgNO_3_ as the trapping solution. However, we observed spuriously high precipitates in the trap as soon as the reducing solution was heated, and the trapping solution turned completely dark in a few minutes, even when there was no sulfate added to the reducing solution. At the same time, we noticed an apparent silver mirror on the inside wall of the collection tube, indicating reduction of Ag^+^ to Ag^0^. This severe reduction of the AgNO_3_ solution is probably due to the high production of PH_3_ from our reducing solution. Different from the conventional reducing solution, our reducing solution used NaH_2_PO_2_ instead of H_3_PO_2_/H_3_PO_3_. NaH_2_PO_2_ starts to decompose and produce PH_3_ at 90°C, while H_3_PO_3_ effectively decomposes to yield PH_3_ at 200°C. Therefore, at the temperature of the reduction experiment (i.e., 124°C), our reducing solution was presumably producing much more PH_3_ than the conventional reducing solution. To remove the excess precipitate other than Ag_2_S caused by PH_3_, we used 1 M HNO_3_ followed by 1 M NH_4_OH to wash the precipitate formed in the AgNO_3_ trapping solution. Only part of the precipitate was removed after these treatments and there was still more precipitate than expected. Thus, AgNO_3_ is not a good choice as the trapping solution, as least for our reducing solution.

To avoid the reduction of Ag^+^ by PH_3_, we next tested 0.1 M Cd(CH_3_CO_2_)_2_ as the trapping solution and following the strategies described in Forrest et al.^17^ Despite this, excess precipitation was still frequently observed after AgNO_3_ was added to the trapping solution for conversion of CdS into Ag_2_S. In particular, we noticed that during the collection of H_2_S, yellow material was accumulating at the wall directly above the surface of the Cd(CH_3_CO_2_)_2_ solution, indicating the formation of CdS. However, at the same time, the entire Cd(CH_3_CO_2_)_2_ solution became light brown and the brown color became deeper with increasing trapping time. When AgNO_3_ was added after the collection, the trapping solution turned dark with extensive precipitate at the same time. Obviously, there were still interferences between the trapping solution and/or AgNO_3_ with the volatile product(s) of the reducing solution. Similarly, excess precipitate remained after washing with 1 M HNO_3_ and 1 M NH_4_OH. This, together with the toxic nature of Cd^2+^, made us decide to abandon Cd(CH_3_CO_2_)_2_ as the trapping solution in our system.

**Figure 3:**
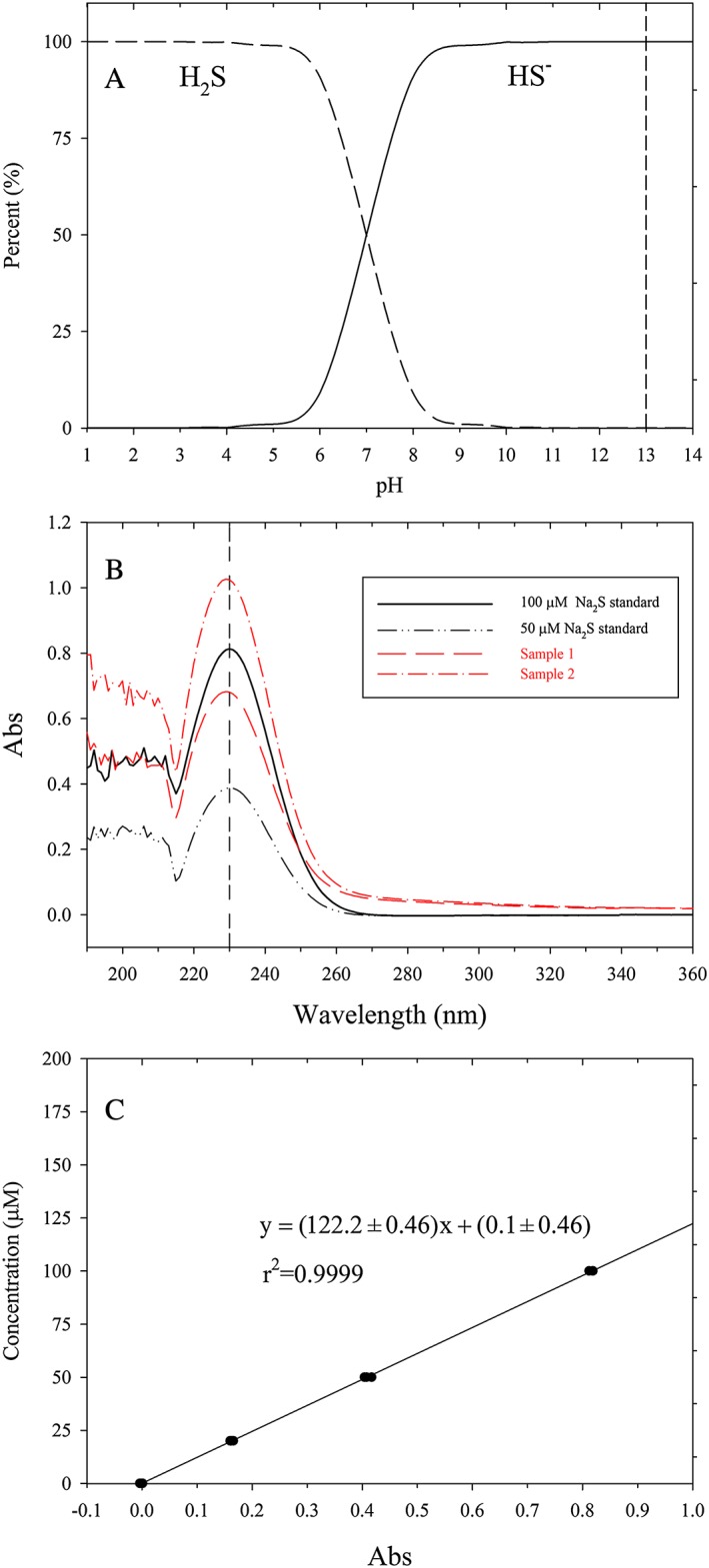
A) Percentages of H_2_S and HS^‐^ in solutions with different pH calculated with pK_a1_ of 7.0 and pK_a2_ of 19, where the vertical dashed line indicates the pH of the trapping solution used in this study. B) Absorbance spectra of Na_2_S working standards and trapping solutions after 1 h collection, where the vertical dashed line indicates the absorbance peak of 230 nm; C) A 3‐day averaged calibration curve for H_2_S quantification [Color figure can be viewed at http://wileyonlinelibrary.com]

Instead, we used 0.1 M NaOH as the trapping solution to collect H_2_S. At a pH of 13, the trapped H_2_S mainly existed in the form of HS^−^, as shown in Figure 3A. Since the NaOH trapping solution was purged with He, under this condition the dissolved O_2_ concentration was very low and thus the trapped sulfide was stable. The use of NaOH as the trapping solution has two advantages: (1) the trapped H_2_S can be precisely quantified in real‐time using UV spectrophotometry, as described in section 2.4, and thus the progress toward to complete reduction of a sulfate sample can be monitored; and (2) no reaction occurs between PH_3_ and AgNO_3_ thus avoiding the production of excessive mass interference.

After sample collection, 1 mL 0.01 M AgNO_3_ was added to the trap to produce Ag_2_S. AgOH was produced at the same time, and this quickly changed to Ag_2_O. The suspension was allowed to settle for 10–20 min after thorough shaking, and a few drops of 68% HNO_3_ were then added to acidify the trapping solution. Ag_2_O was readily dissolved in the acidified solution and only Ag_2_S remains.

### 3.2 H_2_S yield

In the 0.1 M NaOH trapping solution, sulfide was mainly present in the form of HS^−^ (Figure 3A). Figure 3B shows the typical absorbance spectra of two Na_2_S working standards (in 0.1 M NaOH matrix) and two NaOH trapping solutions after 2 h collection of H_2_S and, as expected, the absorbance spectra peak was at ~230 nm, consistent with that from Guenther et al.^22^ Figure 3C shows the plot of the average of the calibration curve over 3 days of analyzing working standards.

**Figure 4:**
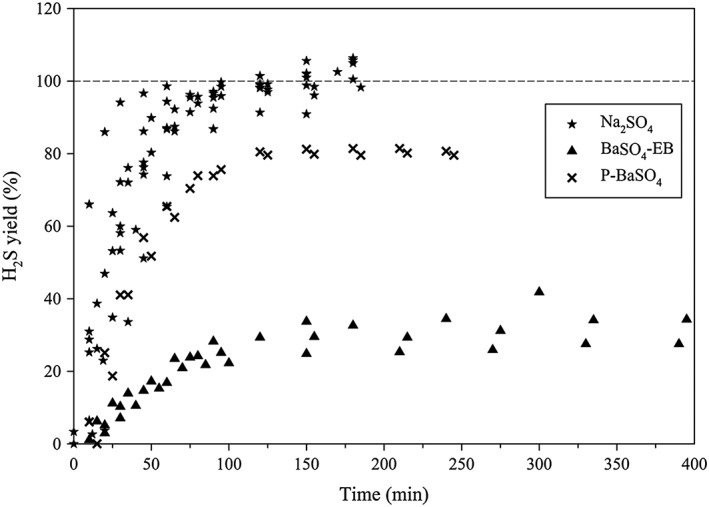
Time‐resolved yields of H_2_S from the reduction of dry Na_2_SO_4_, BaSO_4_‐EB (BaSO_4_ with excess Ba^2+^) and P‐BaSO_4_ (pure BaSO_4_)

As described in section 2.3, three different sulfate samples were processed using our system, Na_2_SO_4_, BaSO_4_‐EB and P‐BaSO_4_, and the time‐resolved H_2_S yields from these three materials are plotted in Figure 4. The real‐time production of H_2_S was monitored by UV determination of HS^−^ in the trapping solution every 15–20 min. Once the produced H_2_S reached the amount expected from the starting sulfate, or no longer increased with time, the block heater was turned off and the reduction train was flushed with He for a further 20 min after the reaction tube had cooled to room temperature.

In general, Na_2_SO_4_ was reduced faster than P‐BaSO_4_, and much faster than BaSO_4_‐EB. Regardless of the quantity of the starting sulfate, after 1 h of reduction an average H_2_S yield of 85.7 ± 10.3% was reached when Na_2_SO_4_ was the starting material. In comparison, the H_2_S yield after 1 h of reduction was 63.9 ± 2.1% for BaSO_4_‐EB and only 18.5 ± 0.04% for P‐BaSO_4_. After 2 h, a 99.5 ± 3.7% yield was reached for Na_2_SO_4_, indicating the completion of the reduction. However, after 2 h, it appeared that no more H_2_S was produced for BaSO_4_‐EB and P‐BaSO_4_, and the yield remained at 80.4 ± 0.75% for BaSO_4_‐EB and 28.5 ± 0.09% for P‐BaSO_4_ after 4 or 5 h. For two of the BaSO_4_‐EB samples, we let the reaction continue overnight, and the yield increased from 41.7% and 34.5% at 5 h to 58.3% and 86.5%, respectively.

**Figure 5:**
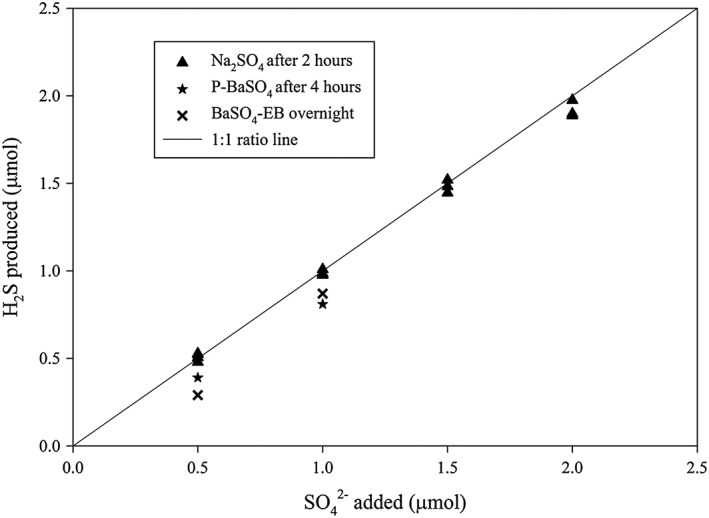
Yields of H_2_S from the reduction of Na_2_SO_4_, BaSO_4_‐EB (BaSO_4_ with excess Ba^2+^) and P‐BaSO_4_ (pure BaSO_4_) at different sulfate quantities at the time that the reaction is stopped

The final yields (yield after stopping the reaction) of Na_2_SO_4_, BaSO_4_‐EB and P‐BaSO_4_ sample with different quantities of sulfate are plotted in Figure 5. Overall, Na_2_SO_4_ was often 100% reduced within 2 h regardless of the starting quantity, even when the drying agent and the cryogenic trap were put in‐line, while a 100% yield for BaSO_4_‐EB and P‐BaSO_4_ was never observed even after overnight heating.

The different apparent reaction speeds and yields of H_2_S between Na_2_SO_4_, BaSO_4_‐EB and P‐BaSO_4_ and the reducing solution probably reflect the effect of the sulfate salt solubility. Na_2_SO_4_ is soluble in water, while BaSO_4_ has a very low solubility of 0.01 μmol/mL in water at 20°C and less than 0.02 μmol/mL at ~120°C.^25^ Given the small volume of the reducing solution used (1 mL), there would be less than 2% of the added BaSO_4_ (if 1 μmol is added) dissolved. Our observations clearly point to the sulfate ion (SO_4_^2−^) or sulfuric acid (H_2_SO_4_) as the reactive species with the reducing acids, i.e. the sulfate salt has to be dissolved first in order to produce H_2_S. This explains why the BaSO_4_ samples reacted so slowly with the reducing solution relative to Na_2_SO_4_. In addition, if there is an excess of Ba^2+^ ions in the solution (due to the dissolution of the excess BaCl_2_ used to precipitate BaSO_4_ from Na_2_SO_4_), this will inhibit the dissolution of BaSO_4_ as the dissociation equilibrium of BaSO_4_ will be pushed to the BaSO_4_ side, following Le Chatelier's principle. This probably explains why the reducing reaction with P‐BaSO_4_ was faster than that with BaSO_4_‐EB. To confirm the effect of excess Ba^2+^ ions on the reduction of BaSO_4_, we prepared a few BaSO_4_ samples with considerably more Ba^2+^ by adding 1 mL of 0.1 M BaCl_2_ to 1 mL of 1 mM Na_2_SO_4_ solution. These samples were then directly dried without removing the supernatant from the precipitate. For these samples, after the reduction started, we measure the trapping solution every hour for 7 h, and no H_2_S was detected at any time.

Therefore, the solubility of the sulfate salt largely affects the reduction speed and the overall yield. We thus recommend extracting and converting sulfate in natural samples into Na_2_SO_4_ whenever possible when applying our reducing solution. The extraction of sulfate can be conducted using the IC method described in Geng et al^26^ or the anion‐exchange resin method described in Le Gendre et al.^21^ If the use of BaSO_4_ is unavoidable, excess Ba^2+^ should be removed after precipitating BaSO_4_ with BaCl_2_, while increasing the volume of the reducing solution (e.g., using 10 mL instead of 1 mL) and/or the reaction time may improve the yield.

### 3.3 Isotope analysis of the standard materials

**Figure 6:**
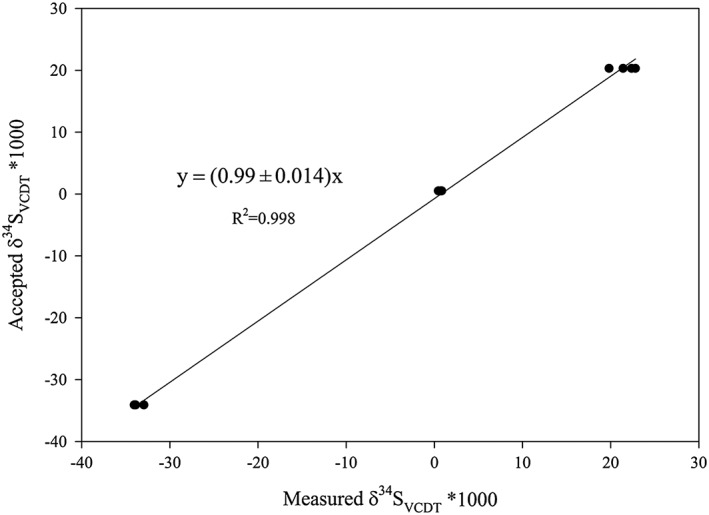
Measured versus accepted δ^34^S (‰)_VCDT_ values of IAEA‐SO‐5, IAEA‐SO‐6 and NBS‐127. The reduction of these sulfate standards to H_2_S were conducted using the protocol described in this study

Since the overall goal of reducing sulfate to sulfide is to perform the four‐sulfur isotopes analysis, we processed three different barium sulfate standards, IAEA‐SO‐5, IAEA‐SO‐6 and NBS‐127, which were equivalent to P‐BaSO_4_ samples. Unfortunately, there are no international standards in sodium sulfate form and thus a strict comparison of the isotopic precision of the reduction step for the two chemical forms is impossible. Even a simple comparison of the salt from an identical sulfate batch is not possible, as BaSO_4_ reduction will never reach full decomposition. The fluorination yields from Ag_2_S to SF_6_ and sulfur isotopic compositions measured from these standards are listed in Table 1. The fluorination yield range is from 84.6 to 113.5% with an average of 101 ± 7.5%, except for one standard with a low yield of 26.1%. Regardless of the fluorination yield, the measured isotopic ratios of all the processed sulfate standards are statistically consistent with their accepted values, including the one with relatively low yield (26.1%). The measured δ^34^S(‰)_VCDT_ values of all standards with different quantities of sulfur (0.34–2 μmol in SF_6_) versus their accepted δ^34^S(‰)_VCDT_ values are plotted in Figure 6. A least‐squares linear regression gives a slope of (0.99 ± 0.01), suggesting good reproducibility and the conservation of sulfur isotopic composition during the reduction of sulfate to sulfide using our reducing system, despite the reduction yields of these standard materials not being 100%. This is not a surprise. In fact, if any sulfur isotope fractionation occurs during the reduction, it would be between the solid BaSO_4_ and the dissolved HSO_4_^−^ (the form of SO_4_^2−^ in concentrated acid solution), but not in the step(s) from SO_4_^2−^ to H_2_S because the dissolved part is 100% converted into H_2_S. Kusakabe and Robinson^27^ found that the sulfur isotope fractionation between solid BaSO_4_ and the dissolved HSO_4_^−^ in the BaSO_4_–HSO_4_–H_2_O system is very small (less than 0.4‰ in the temperature range from 110 to 350°C), which could explain why the solubility effect seems to not affect the isotopic measurements.

**Table 1:** Fluorination yields and measured isotopic ratios of the sulfate standards processed with this system

Standards	Ag_2_S (mg)	SF_6_ yield (%)	Δ^33^S values vs CDT (‰)		δ^34^S values vs CDT (‰)		Accepted δ^34^S^a^ values vs CDT (‰)
NBS‐127	0.20	101.7	0.015	0.025 ± 0.010	19.8	21.6 ± 1.3	20.3 ± 0.5
	0.10	105.3	0.018		22.4		
	0.08	93.7	0.033		22.8		
	0.12	98.2	0.034		21.4		
IAEA‐SO‐5	0.51	104.6	0.063	0.097 ± 0.071	0.7	0.7 ± 0.2	0.5 ± 0.5
	0.52	101.6	0.052		0.7		
	0.82	*26.1*	*0.203*		0.8		
	0.21	99.3	0.067		0.5		
IAEA‐SO‐6	0.41	113.5	0.077	0.086 ± 0.020	‐34.0	‐33.5 ± 0.6	‐34.1 ± 0.5
	0.46	106.9	0.065		‐33.9		
	0.13	102.0	0.110		‐32.9		
	0.15	84.6	0.090		‐32.9		

The values of Δ^36^S are not reported as when the samples were measured the mass spectrometer had a high background of mass 131 (15 to 50 mV) and thus the Δ^36^S data were discarded. The initial masses of the BaSO_4_ standards were only approximately weighed, and the mass(es) in Ag_2_S form were obtained according to the measured H_2_S production after ~5 h of reduction.

a Accepted values are taken from Halas and Szaran.^28^

For these standards, we also reported the Δ^33^S values and they are all not distinct from what can be expected from mass‐dependent fractionation. However, we did not include the Δ^36^S values as, when these standards were measured, the mass spectrometer had a high and variable background at *m/z* 131 up to 50 mV which caused the δ^36^S values to drift and made them unreliable.

## 4 CONCLUSIONS

We present a simple and reliable reducing method modified from the literature for the conversion of sulfate into sulfide for four‐sulfur isotopes analysis. This system is simple to set up, easy to replace and cheap to acquire and is made from sealed test tubes and PEEK flow lines (metal part, e.g. needle, in contact with the hot reducing solution is not allowed). This method uses a reducing solution made of 100 mL 57% HI and 13 g NaH_2_PO_2_, and a very small amount (1 mL) of reducing solution was demonstrated to be able to completely reduce a soluble sulfate salt (0.5–2.5 μmol) to sulfide within 2 h, thus minimizing the use of relatively expensive HI. In practice, nothing prohibits the recycling of the used reducing solution by adding a few mg of NaH_2_PO_2_ in order to reduce I_2_ back to I^−^ in a boiling flask if the used solution turns brown.^14^ In addition, the reduction train avoids the use of a distillation apparatus, and multiple reduction trains can be operated at a time, making it easier to process multiple samples simultaneously. The use of NaOH as the trapping solution allows the assessment of reduction yield directly from UV determination of HS^‐^ in the trapping solution.

This new approach was demonstrated to produce H_2_S very rapidly with a 100% recovery when soluble sulfate salt was used (e.g., Na_2_SO_4_), as opposed to BaSO_4_ for which the kinetic was slow and conversion never reached 100% even after overnight reaction. However, despite the relatively low reduction yield for BaSO_4_, there was no significant isotope fractionation effect induced by the reduction. As it is the dissolved part of the sulfate salt that reacts with the reducing solution, this method is most suitable for natural samples containing soluble sulfate (e.g., aerosol, snow and ice core), which can be extracted (e.g., by the resin method) and converted into Na_2_SO_4_. The use of the barite precipitate method for sulfate extraction and purification is not recommended as the salt solubility inhibits the reduction speed and yield. If BaSO_4_ is the main form of sulfate (e.g., barite), increasing the volume of the reducing solution and/or the reaction time may improve the H_2_S yield although there is no guarantee of a complete conversion. While poor conversion and fluorination yields do not seem to introduce isotope fractionations, poor yield reduces the sensitivity of the method to sample sizes above a few micromoles of sulfate and it may also have consequence on the mass‐dependent slopes between the sulfur isotope ratios as the ^33^S/^32^S ratios of the international standards have never been calibrated.
